# THE FALL OF THE BERLIN WALL AND STEREOTYPES OF AGING: AN INTERGENERATIONAL STUDY ABROAD EXPERIENCE

**DOI:** 10.1093/geroni/igz038.488

**Published:** 2019-11-08

**Authors:** Suzie Macaluso

**Affiliations:** Abilene Christian University, Abilene, Texas, United States

## Abstract

One of the biggest barriers to encouraging a new generation of students to consider careers in aging are the ageist attitudes that they hold and the negative images of aging that they are bombarded with through the media. Sociologists have learned that an effective way to combat ageism is to bring together individuals from different cultural groups, including different age cohorts, to improve social attitudes. In a 2018 AGHE presentation, Jill J. Naar, explored the idea of promoting age-friendly universities by creating intergenerational education tourism programs. In this presentation I share my experience in leading an intergenerational study abroad in Germany that included five generations studying the creation of public memory on the eve of the 30th anniversary of the fall of the Berlin Wall. I will share some of the logistical considerations along with the way that the study abroad helped to meet some of the AGHE competencies for undergraduate programs in Gerontology.

